# A Feasibility Study on the Use of Equine Chondrogenic Induced Mesenchymal Stem Cells as a Treatment for Natural Occurring Osteoarthritis in Dogs

**DOI:** 10.1155/2019/4587594

**Published:** 2019-06-02

**Authors:** Robert Daems, Lore Van Hecke, Ilona Schwarzkopf, Eva Depuydt, Sarah Y. Broeckx, Michael David, Charlotte Beerts, Peter Vandekerckhove, Jan H. Spaas

**Affiliations:** ^1^Dierenartsencentrum Malpertuus, 9070 Heusden-Destelbergen, Belgium; ^2^Global Stem Cell Technology NV, 9940 Evergem, Belgium

## Abstract

Conventional treatments of osteoarthritis (OA) reduce pain and the inflammatory response but do not repair the damaged cartilage. Xenogeneic peripheral blood-derived equine chondrogenically induced mesenchymal stem cells (ciMSC) could thus provide an interesting alternative. Six client-owned dogs with confirmed elbow OA were subjected to a baseline orthopedic examination, pressure plate analysis, general clinical examination, hematological analysis, synovial fluid sampling, and radiographic examination, and their owners completed two surveys. After all examinations, a 0.9% saline solution (placebo control product = CP) was administered intra-articularly. After 6 weeks, all examinations were repeated, owners again completed two surveys, and equine ciMSCs were administered in the same joint. After another 6 weeks, dogs were returned for a final follow-up. No serious adverse events or suspected adverse drug reactions were present during this study. No significant differences in blood analysis were noted between the CP and ciMSC treatment. Two adverse events were observed, both in the same dog, one after CP treatment and one after ciMSC treatment. The owner surveys revealed significantly less pain and lameness after ciMSC treatment compared to after CP treatment. There was no significant difference in the orthopedic examination parameters, the radiographic examination, synovial fluid sampling, and pressure plate analysis between CP treatment and ciMSC treatment. A single intra-articular administration of equine ciMSCs proved to be a well-tolerated treatment, which reduced lameness and pain according to the owner's evaluations compared to a placebo treatment.

## 1. Introduction

Conventional pharmacological treatments of osteoarthritis (OA) consist of non-steroidal anti-inflammatory drugs (NSAIDs) and/or corticoids. These pharmaceuticals reduce pain and the inflammatory response, but do not repair the inflicted damage to the cartilage [1–3]. Therefore, adult mesenchymal stem cells (MSCs) are an interesting alternative to treat OA, since they can differentiate into different cell lineages, promote the migration of endogenous repair cells, and have an immunomodulatory and immunosuppressive effect [1, 2, 4]. In humans, several studies have been performed using autologous MSCs to treat OA, with overall positive results on safety and efficacy [5]. Similarly in dogs, several studies describe promising results using autologous adipose-derived MSCs [6–10]. However, for treatment purposes, allogeneic or xenogeneic MSCs are a more appealing option, since these preclude the need of harvesting tissues of each individual patient and eliminate the prolonged period needed for the production of the “ready-to-treat” end product [2, 5, 11, 12]. Moreover, the quantity and quality of MSCs decline with the increasing age of the donor [1, 13], which poses a problem as OA becomes more prevalent with aging. Thus, allogeneic or xenogeneic transplantation of MSCs could address this problem, since this allows for a stringent selection of healthy and high qualitative donors. Xenogeneic MSCs are interesting in particular, as tissue harvesting from readily available healthy donor animals could provide a very cost-effective solution to produce MSCs for human applications. Especially, as the MSC source is minimally invasive such as peripheral blood, so harvesting can be performed repeatedly with minimal donor site morbidities and little discomfort for the animal. Additionally, for dogs specifically, their MSCs have a low potential for commercial applications, since canine MSCs display a reduced culture capacity and early senescence compared to human and equine MSCs [14–16]. Another advantage of using xenogeneic MSCs is the absence of the risk of transferring highly virulent species-specific pathogens associated with transfusing blood or plasma-derived products (hepatitis B in humans, canine distemper virus in dogs, etc.) [17, 18].

The feasibility and safety of the use of xenogeneic MSC for treating various diseases have been increasingly investigated [12, 19, 20]. Since canine models are regularly used for human OA [21], valuable information on xenotransplantation of MSCs can be obtained from animal studies using dogs. However, to date, the use of peripheral blood-derived MSCs to treat OA in dogs has not been tested. Moreover, the environment in a degenerated joint might not provide the correct stimulus for MSC differentiation and signaling or, alternatively, may even negatively influence their functionality. Therefore, a chondrogenic induction of MSCs before treatment may improve the clinical outcome. Based on recent studies, the intra-articular administration of peripheral blood-derived allogeneic chondrogenic induced MSCs (ciMSCs) in horses effectively reduces lameness and joint effusion, yields some chondroprotective effects, and provides a safe treatment for mild to moderate joint inflammation [22–24]. Therefore, a study was performed to evaluate the feasibility of a single intra-articular administration of equine peripheral blood-derived ciMSCs as a treatment for OA in dogs. It was hypothesized that xenogeneic administration of ciMSCs would provide a safe treatment and would effectively reduce lameness. The results of this study will also provide some preliminary information on the feasibility of this treatment for the application in humans.

## 2. Materials and Methods

### 2.1. Study Design and Eligibility Criteria

This study was a prospective placebo-controlled clinical study and was approved by the local ethics committee of Global Stem Cell Technology (approval number EC_2016_002; permit number LA1700607). Additionally, the study was conducted according to applicable European and national regulatory requirements and in compliance with Directive 2010/63/EU. An informed consent was obtained from all the owners. All patients with suspected OA of the humeroradial joint admitted to the clinical study site were screened for eligibility in this study. Dogs were enrolled in this study if they presented stable pain and lameness associated with unilateral or bilateral OA of the humeroradial joint lasting for over 1 month. The OA had to be confirmed based on a clinical examination, including a lameness examination, and a radiographic examination according to guidelines of the international elbow group [25]. The animals had to weigh ≥ 10 kg and had to be older than 6 months. Animals were excluded from the study if they showed signs of a systemic disease (based on a general clinical examination and general hematological analysis) or if they presented signs of a disease(s) that could affect the gait (e.g., neurological disorder) prior to enrollment. Pregnant bitches were not allowed in the study. NSAIDs were allowed during the study period if dogs started treatment at least 7 days prior to day 0 and remained on the treatment on the same dose during the entire study period of 12 weeks. Corticosteroids were not allowed during the study period or within 60 days before the start of the study. Any intra-articular treatments or the administration of antibiotics were not allowed during the study period.

### 2.2. Isolation and Chondrogenic Induction of Mesenchymal Stem Cells

The equine chondrogenic induced mesenchymal stem cells (ciMSCs) were produced according to GMP guidelines in a GMP-certified site (nr. BE/GMP/2016/069) as described earlier [24]. Briefly, MSCs were isolated from the peripheral blood collected from the vena jugularis from a single donor horse using a proprietary method. The blood collection of the donor horse was approved by the local ethics committee of Global Stem Cell Technology NV (EC_2012_001 and 2016_003), and the blood was tested for several equine pathogens at Böse laboratory (Harsum, Germany). Isolated MSCs were cultured until passage 5, after which they were characterized as described previously (i.e., determination of cell viability, morphology, presence or absence of cell surface markers, trilineage differentiation, and population doubling times) and frozen [14]. Cells were subsequently thawed, cultured, and chondrogenically induced from passage 9 to passage 10, using a proprietary method and proprietary cell culture medium containing cartilage-inducing growth factors. The cells were characterized by evaluating the gene expression of a chondrogenic marker (cartilage oligomeric matrix protein (COMP)), the presence of cell surface markers (MHC II, CD44, and CD90), the total cell number, population doubling time, viability, and sterility. After chondrogenic induction, ciMSCs were detached, resuspended in 1 mL of Dulbecco's modified Eagle medium low glucose with 10% dimethyl sulfoxide (DMSO, Sigma-Aldrich, Overijse, Belgium) at a concentration of 2 × 10^6^ cells per mL and frozen at −80°C in cryovials until clinical application.

### 2.3. Treatment Allocation

At day 0, all dogs enrolled in this study were first treated intra-articularly in their most severely affected humeroradial joint with a placebo control product (CP) (0.5 mL of 0.9% saline solution). Six weeks later (week 6), after the dogs underwent all examinations and the owner surveys were collected, the ciMSCs were thawed and 0.5 mL was administered intra-articularly (1 × 10^6^ cells) in the same humeroradial joint. Six weeks after ciMSC treatment (week 12 = study end), dogs were brought back for a final follow-up, by which they underwent all examinations and the owners completed the owner surveys. With both intra-articular administrations, the dogs received cimicoxib (2 mg/kg, tablet) PO once a day with the first 7 days as a concomitant treatment to minimize inflammation after the injection. In the first 10 days after treatment, the dogs were also subjected to home confinement and leash walking.

### 2.4. General Clinical Examination and Hematological Analysis

At day 0, week 6, and week 12, all dogs underwent a general clinical examination, consisting out of an evaluation of rectal temperature, heart rate, respiratory rate, mucosal membrane color, capillary refill time, body condition score, hydration, mentation, and behavior of the dog. Additionally, the blood was collected for a hematological and biochemical analysis (Table 1). Owners were asked to monitor their dog daily for (serious) adverse events and suspected drug reactions and, in case of the presence of an adverse event, to return for a clinical examination of the dog.

### 2.5. Pressure Plate Analysis

Before treatment at the start of the study, at week 6 before ciMSC administration and at week 12, all dogs underwent an objective lameness examination using an instrumented treadmill with an integrated pressure plate (GAIT4Dog treadmill, GAIT4Dog, Franklin, New Jersey, USA). Dogs were walked on the treadmill with a velocity between 1.5 and 4.5 km/h depending on the dog's size and temperament. The following parameters were recorded using the dedicated GAIT4Dog software program: stance time (the time elapsed between the first contact and the last contact of one identified paw in sec), stride length (the distance in cm between the heel points of two consecutive paw prints of the same paw), reach (the distance in cm from the heel center of the hind paw to the heel center of the previous forepaw on the same side), stance % (the percentage of stance time compared to stride time), pressure % (the integrated pressure over time for a paw expressed as a percent of the overall integrated pressure over time), and the GAIT4Dog lameness score (GLS). With the GLS system, number 100 presents a normal pressure distribution over the paw of interest and a higher or lower number represents a reduced or increased loading of that paw, respectively.

### 2.6. Orthopedic Examination

At day 0, week 6, and 12, an observational gait analysis was performed using the score system as depicted in Table 2. Additionally, the range of motion of the injected humeroradial joint was determined using a goniometer and a score system (Table 2). Articular pain, joint effusion, and the impact of the lameness on the clinical condition were also scored as presented in Table 2.

### 2.7. Synovial Fluid Sampling

At baseline (day 0), at week 6 (before ciMSC injection), and at week 12, synovial fluid was collected, which was checked for hemarthrosis and scored for viscosity (Table 2).

### 2.8. Radiographic Examination

At day 0, week 6, and week 12, radiographs of both elbow joints were taken according to International Elbow Working Group guidelines [25]. Briefly, two mediolateral projections and one craniocaudal projection of each humeroradial joint were taken. One mediolateral projection was taken with the humeroradial joint in flexion (30-40 degrees) and one with the humeroradial joint in neutral position (100–120 degrees). The craniocaudal projection was taken with a 10-degree pronated rotation. The joints were scored for the presence of OA using the following score system: 0 = normal elbow joint, no evidence of incongruency, sclerosis, or arthrosis (no OA); 1 = presence of osteophytes < 2 mm high, sclerosis of the base of the coronoid process—trabecular pattern still visible (mild OA); 2 = presence of osteophytes of 2-5 mm high, obvious sclerosis (no trabecular pattern) of the base of the coronoid processes (moderate OA); and 3 = osteophytes of over 5 mm found anywhere in the joint (severe OA). Additionally, the presence of the following primary lesions was noted: malformed or fragmented medial coronoid process (FCP), ununited anconeal process (UAP), osteochondrosis of medial aspect of humeral condyle, incongruity of articular surface (INC) (step of >5 mm between radius and ulna), and calcification in the soft tissue.

### 2.9. Owner Surveys

At day 0, week 6, and week 12, owners were asked to complete two owner surveys. One survey was created based on the study of Hudson et al. [26] and consisted out of 11 questions using a visual analogue scale of 10 cm. One question gauged the owners' overall evaluation of the dog's pain and life quality. Three questions concerned the dog's mood, three concerned the dog's amount of activity, two concerned the dog's stiffness, and two concerned the dog's pain. The left side of the visual analogue scale corresponded with the “worst-case” scenario (very bad mood of the dog, very stiff, very lame, etc.), and the right side of the visual analogue scale corresponded with the “best case” scenario (very good mood, no stiffness, not lame, etc.). The second survey consisted of the validated canine brief pain inventory (CBPI) [27]. This survey consists of two parts. One part gauges the owner's evaluation of the dog's pain at its worst, at its least, on average, and right now on a scale from 0 to 10, with 0 representing “no pain” and 10 representing “extreme pain.” The mean of these 4 scores was calculated to obtain the pain severity score (PSS). The second part of the survey evaluates the interference of the dog's pain with its general activity, its enjoyment of life, and its ability to rise, to walk, to run, and to climb stairs again on a scale of 0 to 10 with 0 being “no interference” and 10 being “completely interferes.” The mean of these 6 scores was calculated to obtain the pain interference score (PIS). The CBPI also includes a separate question on the owner's assessment of the quality of life of his/her dog on a 5-point scale from “poor” to “excellent.”

### 2.10. Statistical Analysis

All statistical analyses were performed using IBM SPSS Statistics for Windows, version 25.0. Normal distributed continuous data was analyzed using paired sample *t*-tests for comparing the two treatment groups. Normal distribution of the differences of continuous data was checked using Kolmogorov-Smirnov tests and Q-Q plots. Not normally distributed data or ordinal data was analyzed using related sample Wilcoxon signed-rank tests. Binary data was analyzed using related sample McNemar tests. A *P* < 0.05 was considered to be statistically significant.

## 3. Results

### 3.1. Isolation and Chondrogenic Induction of Mesenchymal Stem Cells

The cells at passage 5 displayed all properties of MSCs. They were able to adhere to plastic, a successful trilineage differentiation was achieved, and the cells were positive for CD29 (100%), CD44 (100%), and CD90 (100%) and negative for CD45 (1%) and MHC II (0%). The average population doubling time (PDT) over 10 passages was 1.4. Passage 10 ciMSCs displayed a successful chondrogenic induction (4.4-fold COMP expression), 96% viability, and the presence of an MSC immunophenotype (87% CD44, 98% CD90, and 0% MHC II).

### 3.2. Animals

A total of 6 client-owned dogs were enrolled in this study: 3 intact males and 3 spayed females, aged 5 to 10 years, with a body weight of 18 to 52.5 kg. The dogs belonged to the following breeds: Labrador Retriever (*n* = 2), German Shepherd (*n* = 1), American Staffordshire Bull Terrier (*n* = 1), Rhodesian Ridgeback (*n* = 1), and Dutch Partridge (*n* = 1).

### 3.3. General Clinical Examination and Blood Analysis

No serious adverse events or suspected adverse drug reactions were noted during the entire study period. One dog portrayed occasional nonproductive vomiting during the entire study period, starting 6 days after the CP administration. The dog was treated with 10 days of 40 mg of omeprazole (Omeprazole Sandoz, Sandoz, Vilvoorde, Belgium) starting three days before ciMSC administration. The same dog also portrayed 4 days of diarrhea and vomiting 1 week after the ciMSC administration, when the omeprazole treatment was ended. For this event, the dog received a complementary feed for 5 days, consisting of electrolytes, methylsulfonylmethane, and cranberry (Protectdiar forte, tablets, Ecuphar, Oostkamp, Belgium) and probiotics for 7 days (Fortiflora, Purina, Brussel, Belgium). Both adverse events were presumably due to the concomitant NSAID administration after the intra-articular treatment administration of study products.

None of the parameters measured on the clinical examination was significantly different between the CP evaluation point (week 6) and the ciMSC evaluation point (week 12). There were also no significant differences in parameters measured during the hematological and biochemical analysis of the blood between the two treatments.

### 3.4. Pressure Plate Analysis

**Figure 1:**
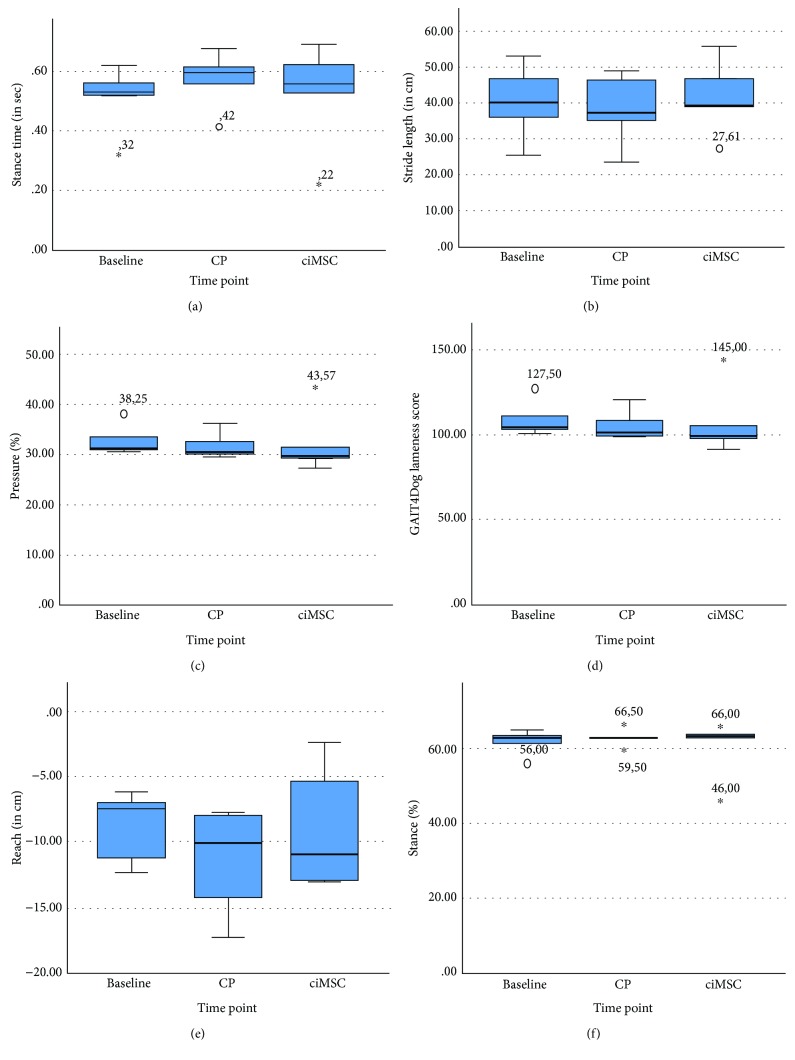
Box plot representation of (a) the stance time, (b) the stride length, (c) the pressure %, (d) the GAIT4Dog lameness score, (e) the reach, and (f) the stance % measured with the pressure plate analysis at baseline (day 0), after placebo control product (CP) administration (week 6) and after xenogeneic chondrogenic induced mesenchymal stem cell (ciMSC) administration (week 12). No significant differences were noted in any of the parameters between CP treatment and ciMSC treatment.

There was no significant difference in any of the parameters measured with the pressure plate analysis between CP treatment and ciMSC treatment (Figure 1).

### 3.5. Orthopedic Examination

**Figure 2:**
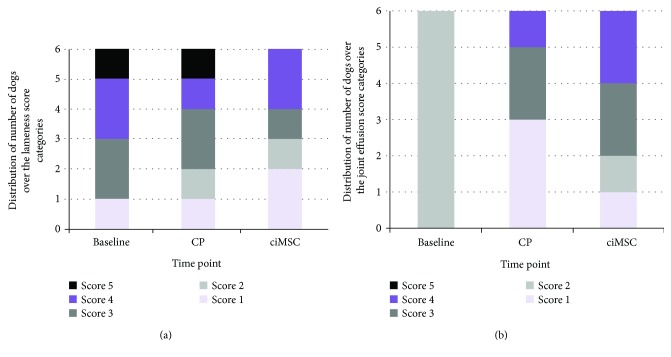
Distribution of the number of dogs over the different (a) lameness score and (b) joint effusion score categories at baseline (day 0), after placebo control product (CP) administration (week 6) and after xenogeneic chondrogenic induced mesenchymal stem cell (ciMSC) administration (week 12).

Despite the observed improvement in lameness scores after the ciMSC treatment when the data was examined descriptively (Figure 2(a)), the difference between the treatment groups was not significantly different (*P* = 0.083). Additionally, when the data was examined descriptively, joint effusion seemed to increase after both CP and ciMSC administrations (Figure 2(b)). However, no significant difference was seen between the treatment groups (*P* = 0.564). The other parameters, i.e., the range of motion in degrees and scores, articular pain, and impact on clinical condition, were also not significantly different between the treatment groups.

### 3.6. Synovial Fluid Sampling

Hemarthrosis was present in 2 animals at baseline, in 4 animals after CP treatment (week 6 before ciMSC administration), and in 2 animals after ciMSC treatment (week 12). The differences between the CP and MSC treatment were not statistically significant. The viscosity of synovial fluid was also not significantly different between the treatment groups.

### 3.7. Radiographic Examination

At baseline, 1 dog was scored with mild OA based on the radiographic examinations, 3 dogs were scored with moderate OA, and 2 dogs were scored with severe OA. After CP treatment (week 6), 1 dog shifted from moderate OA to severe OA. After ciMSC treatment (week 12), the distribution of the dogs across the score categories remained the same as after the CP treatment.

### 3.8. Owner Surveys

**Figure 3:**
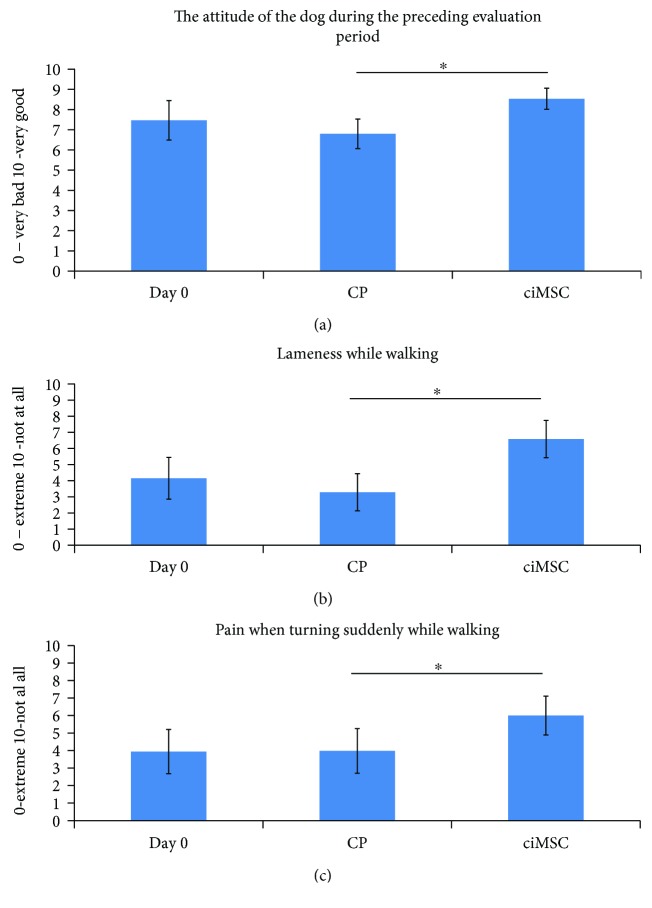
Mean scores (±standard error of the mean) of three questions of the owner survey based on the study of Hudson et al. [26] at baseline (day 0), after placebo control product (CP) administration (week 6) and after xenogeneic chondrogenic induced mesenchymal stem cell (ciMSC) administration (week 12). The asterisk (^∗^) indicates a significant difference in mean scores between the two treatment groups (*P* < 0.05).

Three questions of the owner survey based on the study of Hudson et al. [26] were graded significantly different between CP treatment and ciMSC treatment (Figure 3). The owners scored the attitude of their dog during the preceding month/evaluation period significantly better after ciMSC treatment compared to after CP treatment (*P* = 0.016). The owners also evaluated their dog significantly less lame while walking after ciMSC treatment compared to after CP treatment (*P* = 0.045). Additionally, the owners indicated their dog displayed significantly less pain when turning suddenly while walking after ciMSC treatment compared to after CP treatment (*P* = 0.025).

**Figure 4:**
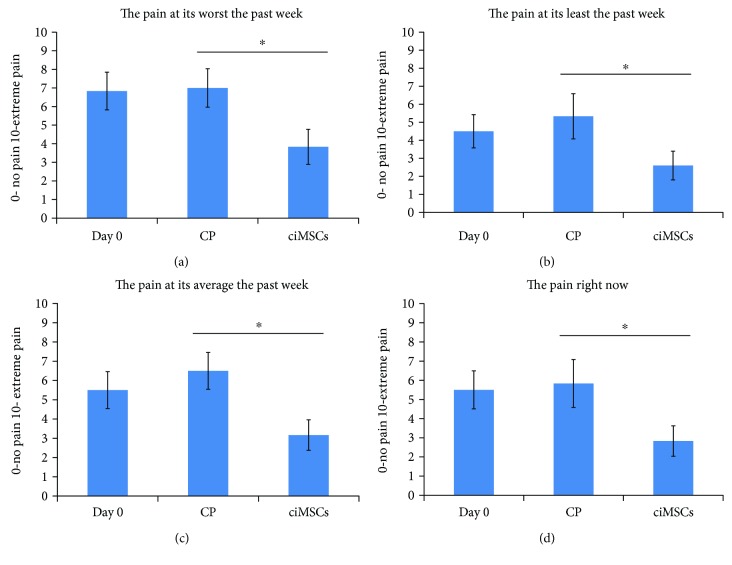
Mean scores (±standard error of the mean) of the questions on pain of the canine brief pain inventory at baseline (day 0), after placebo control product (CP) administration (week 6) and after xenogeneic chondrogenic induced mesenchymal stem cell (ciMSC) administration (week 12). The asterisk (^∗^) indicates a significant difference in mean scores between the two treatment groups (*P* < 0.05).

**Figure 5:**
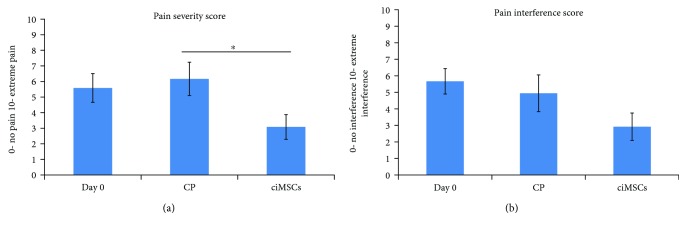
Mean scores (±standard error of the mean) of the pain severity score (PSS) and pain interference score (PIS) at baseline (day 0), after placebo control product (CP) administration (week 6) and after xenogeneic chondrogenic induced mesenchymal stem cell (ciMSC) administration (week 12). The asterisk (^∗^) indicates a significant difference in mean scores between the two treatment groups (*P* < 0.05).

For the canine brief pain inventory survey, several questions were scored significantly different after CP treatment than after ciMSC treatment. The owners scored the pain of their dogs at its least, at its worst, on average, and as it was on the moment of the survey significantly less after ciMSC treatment compared to CP treatment (*P* = 0.04, *P* = 0.008, *P* = 0.007, and *P* = 0.048, respectively) (Figure 4). Additionally, the owners found that the pain interfered significantly less with the ability of the dog to walk and run after ciMSC treatment compared to after CP treatment (*P* = 0.034 and *P* = 0.049, respectively). The PSS was significantly lower after ciMSC treatment compared to after CP treatment (*P* = 0.013) (Figure 5). The PIS was also lower after ciMSC treatment compared to after CP treatment, albeit not statistically significant (*P* = 0.087) (Figure 5).

## 4. Discussion

This study was the first to investigate the feasibility of peripheral blood-derived equine ciMSCs as a treatment for OA in dogs. In line with our hypothesis, equine ciMSC proved to be a well-tolerated treatment for OA in dogs. No suspected adverse drug reactions or serious adverse events were noted. There was however an increase in joint effusion after both CP and ciMSC treatments when the data was examined descriptively. This increase in joint effusion was not significantly different between the treatment groups, and joint swelling has been a reported phenomenon after intra-articular injections in general [28]. Moreover, the effusion was noted 6 weeks after intra-articular administration of the treatments, suggesting the effusion could have another cause, such as an increased activity of the dog. There were two reported adverse events in this study, both presented by the same dog: one after CP administration and one after ciMSC administration. Both adverse events were judged by the investigating veterinarian to be unrelated to the study treatments. Furthermore, the owners of this dog reported previous similar problems outside the study due to long-term use of NSAIDs.

The second part of our hypothesis was partly fulfilled. The administration of ciMSCs significantly reduced lameness, albeit only based on the owner surveys. When the data of the veterinary examinations were examined descriptively, a decrease in lameness was seen after both treatment administrations, with a larger decrease after ciMSC administration. However, this decrease was not significantly different. Since the *P* value was low (0.083), it is possible that the statistical significance would have been reached by including a larger number of dogs in the study. However, it proved to be very difficult to motivate owners to participate in a placebo-controlled study with a cell-based product still in the experimental phase. Moreover, the xenogeneic factor of the study raised additional concerns with the owners about safety and stem cell rejection. Therefore, all dogs enrolled in this study were older dogs who did not respond to conventional treatments, of which the owners considered the ciMSCs as a “last resort treatment.” Since most of these dogs presented moderate to severe OA, profound damage was already present in the joint. Therefore, to observe amelioration in these animals, the effect of the ciMSCs had to be outspoken.

The objective lameness assessment did not reveal any significant difference between the treatment groups. However, it was proved to be difficult to have the dogs walk naturally on the treadmill. Most dogs had the tendency to start pulling the leash or to walk towards the side of the belt to try to lean against the side fences, despite a 10-minute acclimatization period. This resulted in the pressure plate indicating all dogs were lame in both or one hind limb, although they all had a clear history of humeroradial OA. Additionally, one dog was omitted from the objective lameness assessment because of its nervous temperament. Therefore, data of the pressure plate analysis in this study should be interpreted carefully.

The safety in this study was assessed by means of clinical examination and blood analysis. No increase in white blood cell counts, its individual subsets, or globulins after either treatment was noted. However, to get a more profound insight in the safety of ciMSCs as a therapy for OA dogs and on a longer term for humans, functional immunological assays such as mixed lymphocyte reactions or xenoantibody determination should be performed. After all, MHC-mismatched MSCs have been shown to induce alloantibodies in horses [29, 30]. Additionally, xenogeneic MSC administration in horses has been reported to induce an increase of CD4+ cells as determined with mixed lymphocyte reactions [31]. Therefore, it would be interesting to investigate if similar results are seen after the administration of equine ciMSC in dogs.

Although xenogeneic use of MSCs has been described, most studies use humane or porcine MSCs in animal models [12, 19, 32, 33]. Of these studies, Tsai et al. [33] reported on the intra-articular administration of porcine adipose-derived MSCs. Similar to the present study, they found that the owners evaluated an improvement in the dogs' pain and their capacity to perform activities. Additionally, they found no radiographic changes before or after treatment and no allergic reactions. Joint effusion as such was not determined in this study. Tsai et al. [33] also found an improvement in an overall orthopedic score and the peak vertical force and vertical impulse when comparing after treatment values with baseline values. However, they did not include a placebo control and the study consisted of only three cases. Additionally, all statistical analyses were performed per case. Thus, a parameter of the orthopedic examination, a question of the owner questionnaire, or one run of the force plate analysis was considered as an experimental unit. Since an experimental unit should normally be a subject/entity which can receive a treatment independently of the other units, the conclusions in the study of Tsai et al. [33] are to be interpreted with care. A more recent study described the use of equine MSCs in an OA mouse model [34]. They found a chondroprotective effect of the equine MSCs when compared to no treatment. Additionally, they reported the ability of equine MSCs to decrease murine splenocyte proliferation *in vitro*, suggesting an immunomodulatory effect of the stem cells. The present study focused on the clinical effects of xenogeneic administration of equine ciMSCs in client-owned dogs with promising results. However, this precluded the possibility to perform a macroscopic and histologic examination of the joint and to assess possible cartilage regeneration or a chondroprotective effect. Therefore, a follow-up study performed on experimental animals using an OA model is recommended to investigate the histological effects of the equine ciMSCs and perform an in-depth examination if the cells induce an immune response.

The present study has its limitations; it was not blinded and performed on a rather limited number of dogs. Additionally, a full cross-over design (placebo administration followed by ciMSCs and a washout period, followed by a new ciMSC administration and placebo administration) was not possible in the same dogs, as it is not known how long the washout period is for stem cells, if there is one at all. The inclusion of more dogs, as mentioned earlier, was not possible, because of the great reluctant of the owners to participate in a placebo-controlled study containing a stem cell product still in the experimental phase. Therefore, this study has to be considered a pilot study which investigated the feasibility of ciMSC as a treatment for naturally occurring OA in dogs and in humans. Based on this study, the treatment proved to be feasible, but further follow-up studies using experimental dogs are necessary. Further studies are preferably randomized, double blinded, and placebo controlled and could also assess the added value of repeated injections and their safety.

## 5. Conclusion

The single intra-articular administration of equine ciMSCs to treat naturally occurring OA in dogs proved to be a well-tolerated treatment, which reduced lameness and pain according to the owner's evaluations compared to a placebo treatment. Therefore, this study provides the base for further research investigating the mechanisms of action of equine ciMSCs on OA and initiating the development of a new treatment protocol for OA.

## Figures and Tables

**Table 1:** Parameters determined during the hematological and biochemical analysis of blood samples.

Parameter	Unit	Normal reference range
*Hematology*		
Erythrocytes	/L	5.65-8.87 × 10^12^
Hemoglobin	g/dL	13.1-20.5
Hematocrit	%	37.3-61.7
MCV	fL	61.6-73.5
MHV	pg	21.2-25.9
MCHC	g/dL	32.0-37.9
Leucocytes	/L	5.05-16.76 × 10^9^
*Differential blood picture—absolute*		
Neutrophils	/L	2.95-11.64 × 10^9^
Eosinophils	/L	0.06-1.23 × 10^9^
Basophils	/L	0.00-0.10 × 10^9^
Lymphocytes	/L	1.05-5.1 × 10^9^
Monocytes	/L	0.16-1.12 × 10^9^
*Renal function*		
Creatinine	mmol/L	3.89-7.95
Urea	mmol/L	2.5-9.6
*Protein metabolism*		
Total protein	g/L	52-82
*Liver and bile*		
Total bilirubin	*μ*mol/L	0-15
Gamma glutamyltransferase (GGT)	U/L	0-11
Alkaline phosphatase	U/L	23-212
Lactate dehydrogenase (LDH)	U/L	40-400
*Heart and muscle*		
Creatine kinase (CK)	U/L	10-200

**Table 2:** Explanatory overview of the score systems used for the orthopedic examination and synovial fluid sampling.

Parameter	Score	Definition
Lameness assessment	1	Stands, walks, and trots normally
	2	Stands normally, slightly painful gait when trotting
	3	Stands normally, slightly painful gait when walking
	4	Stands normally, evident painful gait when walking
	5	Stands abnormally, evident painful gait when walking

Range of motion	1	No limitation of movement or crepitus
	2	10 to 20 percent decrease in range of motion, no crepitus
	3	10 to 20 percent decrease in range of motion with crepitus
	4	20 to 50 percent decrease in range of motion
	5	More than 50 percent decrease in range of motion

Articular pain	1	None
	2	Mild signs (dog turns head in recognition)
	3	Moderate signs (dog pulls limb away)
	4	Severe signs (dog vocalizes or becomes aggressive)
	5	Dog will not allow palpation

Joint effusion	1	None
	2	Mild signs (only at site of injection)
	3	Moderate signs (mild swelling of entire joint)
	4	Severe signs (severe swelling of entire joint)
	5	Extreme (periarticular swelling)

Impact on clinical condition	1	Not affected
	2	Mildly affected
	3	Moderately affected
	4	Severely affected
	5	Very severely affected

Synovial fluid viscosity	1	Watery, no string
	2	Tacky, string < 0.5 cm
	3	String 0.5-4 cm
	4	String > 4 cm

## Data Availability

The data sets used to support the findings of this study have not been made available because of commercial confidentiality.
